# Phosphorylation of S776 and 14-3-3 Binding Modulate Ataxin-1 Interaction with Splicing Factors

**DOI:** 10.1371/journal.pone.0008372

**Published:** 2009-12-23

**Authors:** Cesira de Chiara, Rajesh P. Menon, Molly Strom, Toby J. Gibson, Annalisa Pastore

**Affiliations:** 1 National Institute for Medical Research, Medical Research Council, London, United Kingdom; 2 Structural and Computational Biology Unit, European Molecular Biology Laboratory, Heidelberg, Germany; Emory University, United States of America

## Abstract

Ataxin-1 (Atx1), a member of the polyglutamine (polyQ) expanded protein family, is responsible for spinocerebellar ataxia type 1. Requirements for developing the disease are polyQ expansion, nuclear localization and phosphorylation of S776. Using a combination of bioinformatics, cell and structural biology approaches, we have identified a UHM ligand motif (ULM), present in proteins associated with splicing, in the C-terminus of Atx1 and shown that Atx1 interacts with and influences the function of the splicing factor U2AF65 via this motif. ULM comprises S776 of Atx1 and overlaps with a nuclear localization signal and a 14-3-3 binding motif. We demonstrate that phosphorylation of S776 provides the molecular switch which discriminates between 14-3-3 and components of the spliceosome. We also show that an S776D Atx1 mutant previously designed to mimic phosphorylation is unsuitable for this aim because of the different chemical properties of the two groups. Our results indicate that Atx1 is part of a complex network of interactions with splicing factors and suggest that development of the pathology is the consequence of a competition of aggregation with native interactions. Studies of the interactions formed by non-expanded Atx1 thus provide valuable hints for understanding both the function of the non-pathologic protein and the causes of the disease.

## Introduction

Ataxin-1 (Atx1) is a 98 kDa protein and a member of the protein family containing polymorphic polyglutamine (polyQ) tracts related to neurodegenerative diseases [1]–[3]. Although clinically distinct, these pathologies are all caused by a common mechanism: when the polyQ tract is anomalously expanded above a threshold which varies for each disease the polyQ carrier protein misfolds and aggregates leading to cellular death. Expansion in Atx1 above 35–42 glutamines is associated with spinocerebellar ataxia type 1 (SCA1), an autosomal dominant neurodegenerative disorder characterized by motor coordination deficits caused by progressive loss of Purkinje cells in the cerebellar cortex and neurons in the brain stem and spinocerebellar tracts.

A causative link between polyQ expansion and the disease process is now generally accepted. However, the importance of other regions of the carrier proteins, the so-called “protein context”, has been increasingly appreciated in the past few years [4]–[6]. At the same time, the concept that SCA1 pathology depends on alteration of native protein interactions, rather than on acquisition of new aberrant interactions mediated by polyQ, has gained growing consensus. Atx1 regions other than the polyQ tract have been functionally and structurally characterized and shown to mediate native protein-protein interactions, and to modulate the process of aggregation and pathogenesis [7]–[14]. A major advance in the process of unraveling the molecular bases of SCA1 pathogenesis was achieved by showing that expansion of the polyQ tract is necessary but not sufficient to cause pathology: expanded Atx1 does not produce cerebellar degeneration if it lacks regions other than the polyQ tract such as a nuclear localization signal (NLS) [15] or the AXH domain [14], or if a serine to alanine mutation prevents phosphorylation at residue 776 [16]. Phosphorylation by Akt kinase of this residue, located at a site remote from the polyQ tract, is also essential for Atx1 binding to the multifunctional regulatory protein 14-3-3 [17]. It was also shown that polyQ expansion of Atx1 differentially affects the function of the protein in the context of endogenous protein complexes. In the context of nuclear interactions for instance, it favours the formation of a protein complex containing SPF45, also known as RBM17 [18], a factor which regulates alternative splicing through interactions with other splicing factors [19]–[21], thus contributing to SCA1 neuropathology via a gain-of-function mechanism. Concomitantly, polyQ expansion attenuates the formation and function of another protein complex containing Atx1/Capicua, contributing to SCA1 via a partial loss-of-function mechanism. These results lead directly to the question of which function of Atx1 is modulated by 14-3-3 and by the other factors and how this is linked to pathology.

With the aim of addressing these questions, we set out to study in more detail the mechanism(s) which determine the Atx1 interactome. We found that Atx1 contains a UHM ligand motif (ULM), previously identified in splicing factors [21], which overlaps both with 14-3-3 binding motif and with the NLS. This region, which comprises S776, mediates Atx1 interaction with the UHM domains, RRM-like motifs exclusively present in pre-mRNA processing factors [22], [23]. We assessed that two nuclear proteins, the constitutive element of the spliceosome U2AF65 and the regulatory factor SPF45, both identified in the pre-spliceosome complex, also known as complex A [24], [25], and previously found in the Atx1 interactome [18], [26], bind the protein *in vitro* through a similar ULM/UHM recognition mechanism [21]–[23]. We show by co-immunoprecipitation and co-localization experiments using native as well as over-expressed proteins, that U2AF65 interacts both with polyQ non-expanded and expanded Atx1. The interaction appeared to have a positive effect on the splicing activity of U2AF65. Finally, we investigated how phosphorylation of S776 modulates interaction and found that, while not significantly changing the affinity for the two UHM proteins, it determines a quantitative shift towards complex formation with 14-3-3. This suggests that the interaction with 14-3-3 has the role of segregating Atx1 in a high affinity complex, thus preventing interaction with UHM domains. We conclude that rather than by a ‘simplistic’ gain- or loss-of-function mechanism, SCA1 pathology is triggered by complex alterations of its normal interactome, some of which even exert a protective role against disease.

## Results

### Atx1 Contains a Sequence Match to the UHM Ligand Motif

**Figure 1:**
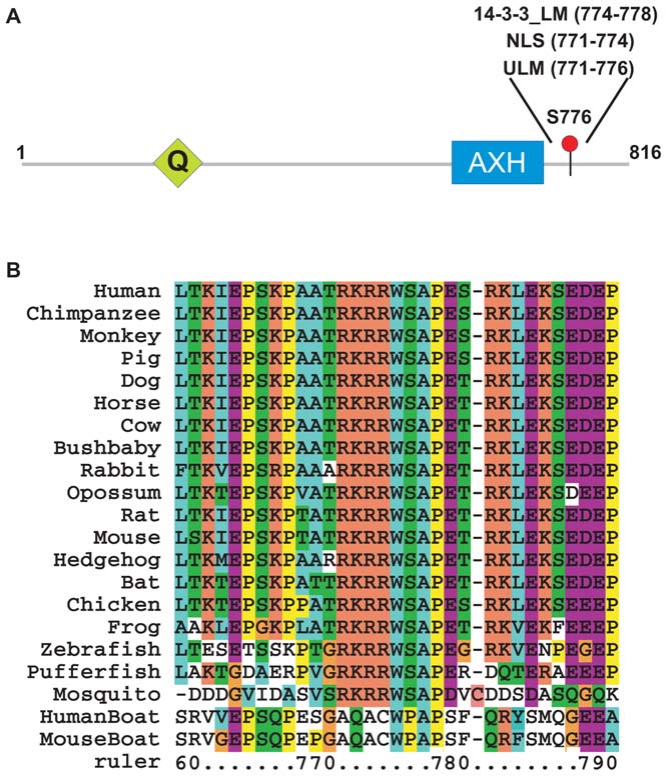
Sequence analysis. A. Schematic representation of the Atx1 architecture. The positions of the polyQ tract (Q) and of the AXH motif are indicated as well as S776. B. Alignment of the ULM motif in Atx1 orthologs and of the corresponding Boat sequences. The alignment was coloured according to standard coding. Sequence numbering follows human Atx1.

In an attempt to shed light on the Atx1 function(s), we analyzed its sequence with the ELM resource for predicting functional short linear motifs in eukaryotic proteins [27], [28]. ELM revealed a short pattern (RKRRWS) in the Atx1 region 771–776 (**Figure 1A**) which gives a perfect match with the UHM ligand motif (ULM). This motif, first identified in the constitutive splicing factors SF1 and SAP155 and known to bind to the UHM domain of U2AF65 [21], is C-terminal to the previously identified AXH domain [8], [9], which is essential for several of the Atx1 functions, including a putative role in transcriptional regulation [10]–[14]. Alignment of Atx1 from different species shows that the ULM match is highly conserved within the Atx1 family but diverges in the Atx1-like Boat protein (**Figure 1B**). This suggests that transcriptional regulation of the AXH domain is uncoupled from the ULM function. Atx1 ULM is absent in *D. melanogaster* and *C. elegans* where Atx1 is very divergent and spans almost exclusively the AXH domain, but is present in the mosquito homologue.

As for most linear motifs [28], [29], ULMs have so far been found only in regions of natively unstructured proteins. The region around this putative motif of Atx1 is predicted as natively disordered [30] (**data not shown**). We concluded from sequence analysis and from the knowledge that Atx1 is also present in the nucleus where splicing occurs, that the match to ULM is a strong linear motif candidate and that, if validated experimentally, it could provide useful insights into the Atx1 function.

The putative Atx1 ULM overlaps with two other motifs. An already identified “mode II” recognition site for the adaptor molecule 14-3-3 (RXXpSXP) is located in the region 773–778 [17], [31]. Also this motif, like Atx1 ULM, includes S776 that has been shown to be phosphorylated by Akt kinase *in cell* and to play a critical role in SCA1 pathogenesis [16]. In addition, the nuclear localization signal (NLS) of Atx1 has been located at 771–774 (RKRR), overlapping with both ULM and 14-3-3 ligand motifs, as the K772T mutant causes a shift in distribution of Q82-expanded Atx1 to the cytoplasm in transgenic mouse and prevents the development of the disease [15].

### Atx1 Recognizes UHM Domains

**Figure 2:**
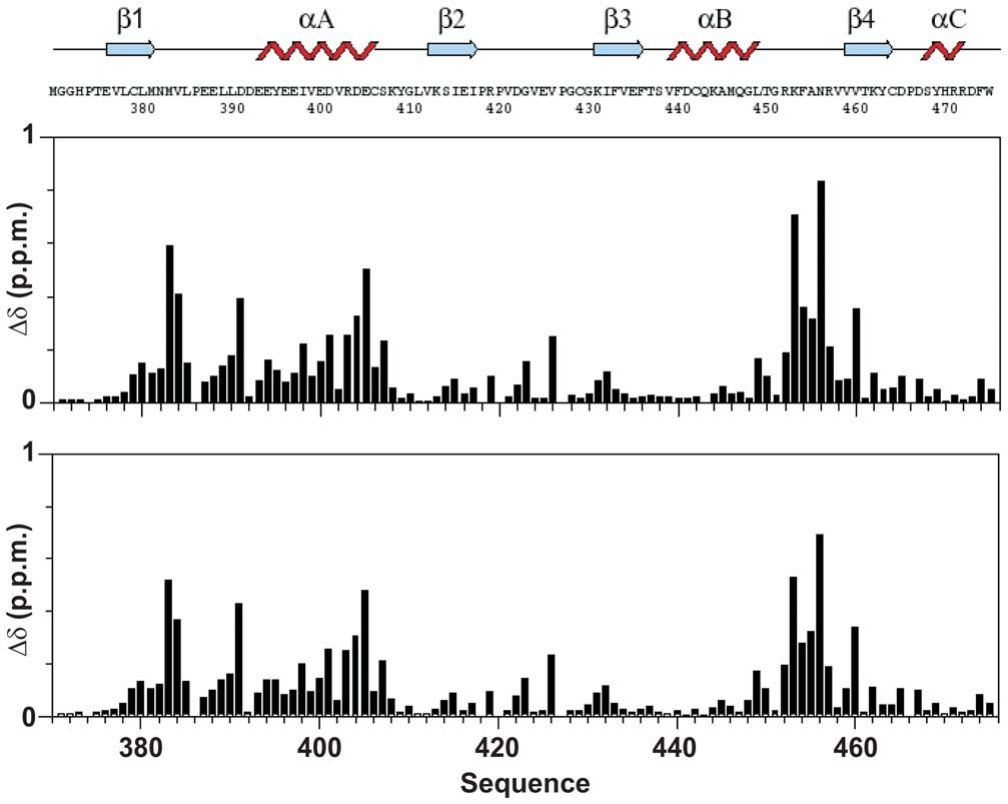
Mapping the effect of Atx1 peptides on U2AF65. Weighted chemical shift perturbation of U2AF65 upon titration with Atx1_S_ULM_PE (upper panel) and Atx1_pS_ULM_PE (lower panel). The U2AF65 sequence and secondary structure elements are indicated.

To assess whether Atx1 recognizes the U2AF65 UHM domain through its putative ULM motif, we performed a titration experiment by NMR and followed the chemical shift perturbation of ^15^N U2AF65 UHM HSQC spectrum upon addition of an unlabelled Atx1 ULM peptide (residues 769–777). The chemical shift variations observed at low ionic strength (30 mM NaCl) are comparable to those observed for SF1 ULM (residues 11–25) under the same conditions [23] suggesting that SF1 ULM and Atx1 ULM bind to U2AF65 UHM in a similar way (**Figure 2**). No significant changes were observed beyond a peptide∶protein equimolar ratio. Disappearance of the spectrum of the free form and appearance of peaks arising from the bound form indicate that Atx1 ULM binds tightly in a slow exchange regime in the NMR time scale, which is indicative of a K_d_ in the nanomolar range for the U2AF65 UHM/Atx1 ULM interaction.

### Phosphorylation of Atx1 S776 Reduces but Does Not Abolish the Interaction with U2AF65 UHM

It has been suggested that, when present in ULM motifs, serine phosphorylation regulates association to UHM [23]. In order to check whether phosphorylation of Atx1 S776 by Akt kinase [17] prevents the interaction with U2AF65 UHM, in analogy with what observed for SF1 S20 [32], we performed a titration experiment by NMR following the chemical shift perturbation of ^15^N labeled U2AF65 UHM upon addition of unlabelled phosphorylated Atx1 ULM (Atx1_pS_ULM) using the same conditions used for the non-phosphorylated peptide. Atx1_pS_ULM showed to bind U2AF65 UHM without appreciable variation of the exchange regime and with minor differences in the values and direction of the chemical shift perturbation as compared to the non-phosphorylated peptide (**Figure 2**).

To quantify even a small weakening of the binding caused by phosphorylation of S776 we measured the dissociation constants (K_d_) by Iso-Thermal Calorimetry (ITC). It has been shown that *in vitro* ULM/UHM recognition is sensitive to the solution ionic strength. To compare directly the K_d_ values with the literature data [21], the ITC measurements were performed at physiological ionic strength (150 mM NaCl) (**Table 1** **and Figure S1**). The affinity decreases by one order of magnitude upon increasing the NaCl concentration, likely as a consequence of an unfavorable entropic effect and in agreement with the expected electrostatic nature of the interaction [21], [23]. Phosphorylation of S776 modulates the Atx1 ULM/U2AF65 UHM interaction by decreasing the affinity approximately three fold.

**Table 1:** Summary of the K_d_'s as obtained from ITC measurements carried out with the different peptides.

Peptide	Sequence	U2AF65_UHM	SPF45_UHM	*14-3-3ζ*
Atx1_S_ULM	769 777 AT**RKRRWS**A	0.2±0.03 µM^a^ 5.6±0.3 µM	n.d.^b^	n.d.
Atx1_pS_ULM	769 777 AT**RKRRWpS**A	0.6±0.08 µM^a^ 18.4±0.7 µM	n.d.	1.0±0.1 µM
Atx1_S_ULM_PE	769 779 ATRK*RRWSAP*E	9.2±0.3 µM	23.4±1.5 µM	n.b.^c^
Atx1_pS_ULM_PE	769 779 ATRK*RRWpSAP*E	35.8±1.3 µM	40.0±1.6 µM	0.4±0.03 µM
Atx1_A_ULM_PE	769 779 ATRK*RRWAAP*E	8.9±0.4 µM	22.5±0.6 µM	n.b.
Atx1_D_ULM_PE	769 779 ATRK*RRWDAP*E	6.4±0.7 µM	0.9±0.07 µM	n.b.

ULM and 14-3-3- binding motifs are underlined on the peptide sequences and marked in bold and italic, respectively.

a 20 mM sodium phosphate buffer (pH 6.4), 30 mM NaCl, 5 mM DTT. Otherwise, 50 mM sodium phosphate buffer (pH 7.0), 150 mM NaCl, 5 mM DTT.

b n.d.: binding not determined.

c n.b.: binding not detectable.

### U2AF65 Interacts with Atx1 *In Vivo*

**Figure 3:**
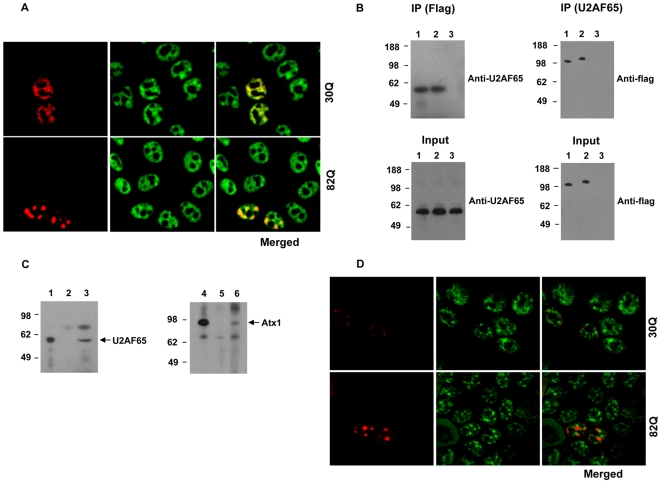
Interaction of Atx1 with U2AF65. A. Co-localization of Atx1 with endogenous U2AF65 in HeLa cells transfected with RFP-tagged Atx1 constructs and analysed by confocal microscopy. Non-expanded (30Q) or expanded Atx1 (82Q) (red) co-localized with endogenous U2AF65 (green), as evidenced by the merged images. B. Interaction of Atx1 with endogenous U2AF65 in HeLa cells. HeLa cells were transfected with flag tagged non-expanded Atx1 (lane 1), expanded Atx1 (lane 2) or empty pCMV constructs (lane 3). Lysates from these cells were immunoprecipitated with anti-flag or anti-U2AF65 antibodies. Pelleted samples were analysed by western blot using anti-U2AF65 and anti-flag antibodies (IP). 5% of the lysates from transfected cells were analysed by western blot using respective antibodies (Input). Molecular weight markers are shown on the left. C. Association of Atx1 with U2AF65 in cerebellum extracts from mouse brain. The extracts were subjected to immunoprecipitation with anti-Atx1 antibodies, anti-U2AF65 antibodies or control anti-flag antibodies. 2.5% of pelleted complexes from anti-U2AF65 IP (lane 1) and 25% from anti-flag IP (lane 2) and anti-Atx1 IP (lane 3) were subjected to PAGE and Western blot analysis using anti- U2AF65 antibodies. 2.5% of pelleted complexes from anti-Atx1 IP (lane 4) and 25% from anti-flag IP (lane 5) and anti-U2AF65 IP (lane 6) were subjected to PAGE and Western blot analysis using anti-Atx1 antibodies. D. Atx1 does not localize with a nuclear speckle marker. HeLa cells were transfected with RFP-tagged non-expanded (30Q) or expanded (82Q) Atx1 (red). Cells were fixed, permeabilized and stained with anti-SC-35 antibodies followed by FITC conjugated secondary antibodies (green). Merged images indicate that neither forms of Atx1 co-localize to the speckles defined by SC-35.

While *in vivo* interaction of Atx1 with SPF45 has been fully demonstrated [18], an interaction with U2AF65 was only suggested by a two-hybrid-screen assay [26]. To test the interaction *in vivo*, we initially performed confocal microscopy co-localization experiments using RFP-tagged Atx1. Analysis of HeLa cells co-expressing the proteins revealed that endogenous U2AF65 and non-expanded Atx1 partially co-localize in the nucleoplasm and in speckle-like structures (**Figure 3A**). PolyQ expanded Atx1 is known to form large nuclear inclusions [10]–[14]. When we analyzed cells co-expressing endogenous U2AF65 and expanded Atx1, U2AF65 was found to localize in the large inclusions formed by expanded Atx1 (**Figure 3A**).

To further confirm the interaction of Atx1 and U2AF65, we then performed co-immunoprecipitation experiments. Lysates from HeLa cells expressing flag-tagged non-expanded or expanded Atx1 were subjected to immunoprecipitation using anti-flag antibodies. Transferring the precipitated complexes onto nitrocellulose membrane and probing with anti-U2AF65 antibody revealed the presence of endogenous U2AF65 in the precipitates (**Figure 3B**). To verify the interaction further, we immunoprecipitated the lysates with anti-U2AF65 antibodies and probed the immune complexes with anti-flag antibodies (**Figure 3B**). The interaction was also verified by co-immunoprecipitation experiments using mouse cerebellum extracts. Immunoprecipitation of the extracts with anti-Atx1 antibody followed by immunoblotting with anti U2AF65 antibody, or immunoprecipitation with anti-U2AF65 antibody and immunoblotting with anti-Atx1 antibody both indicated that endogenous Atx1 and U2AF65 from the extracts associate with each other (**Figure 3C**).

As some of the co-localization appeared to involve the speckle-like structures formed by non-expanded Atx1, we wanted to confirm whether these speckle-like structures are true nuclear speckles where splicing factors are known to accumulate [33]. We observed that the endogenous nuclear speckles formed by a nuclear speckle marker, SC-35, did not co-localize with over-expressed Atx1 (**Figure 3D**). Interestingly, while SC-35 and over-expressed U2AF65 did co-localize in the nucleus, there were speckle-like formations of U2AF65 that were devoid of SC-35 (**Figure S2**).

To address the question of whether interaction with Atx1 influences the cellular functions of U2AF65, we carried out preliminary studies assessing pre-mRNA binding by U2AF65. It was shown recently that forced expression of Sam68, a U2AF65 binding protein, increases CD44 minigene pre-mRNA binding by U2AF65 [34]. In our studies, we assessed CD44 pre-mRNA occupancy by carrying out RNP immunoprecipitation of lysates from cells expressing the two proteins followed by RT-PCR using primers specific for the CD44 minigene. We were able to amplify the CD44 signal from the immunoprecipitates of these cells. However, we observed no significant difference when the cells co-expressed Atx1 (**Figure S3**), suggesting absence of interference.

### Atx1 Has a Positive Effect on U2AF65 Splicing

**Figure 4:**
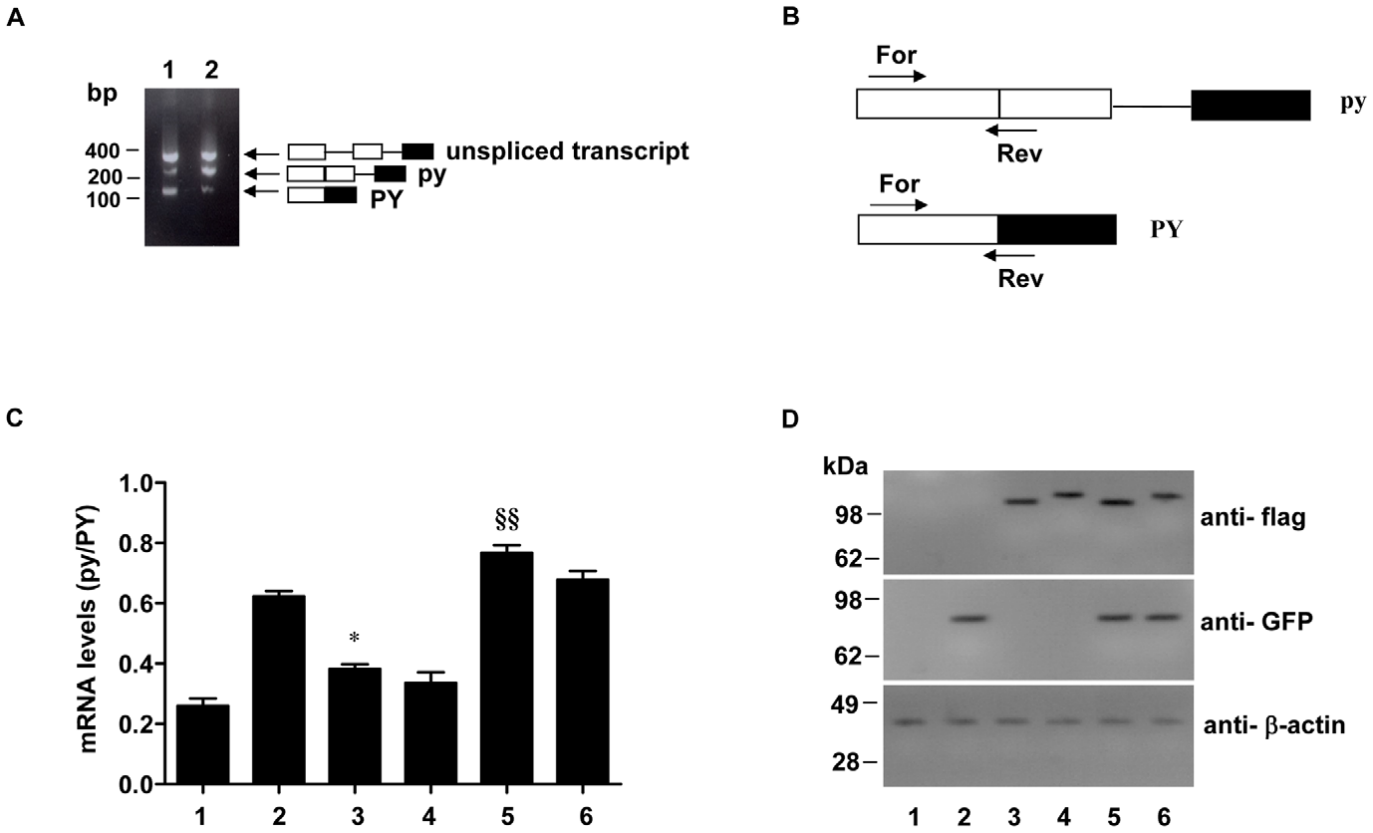
Effect of Atx1 overexpression on alternative splicing of pyPY. A. Alternatively spliced transcripts from cells expressing the pyPY minigene. 293T Cells were transfected with the pyPY reporter minigene plasmid. RNA was isolated 24 h post transfection and RT- PCR analysis was carried out. The predicted alternatively spliced and unspliced primary transcripts are shown on the right by diagrams in which the exons and introns are symbolised by boxes and lines, respectively. Molecular weight markers are indicated on the left. B. Schematic representation of the positions of forward (For) and reverse (Rev) primers used in Quantitative RT-PCR analysis. C. Quantitative RT-PCR analysis of the expression levels of transcripts. 293T cells were transfected with pyPY reporter minigene plasmid together with empty pCMV vector (bar 1), GFP-U2AF65 (bar 2), flag-30Q-Atx1 (bar 3), flag-82Q-Atx1 (bar 4), GFP-U2AF65 and flag-30Q-Atx1 (bar 5) or GFP-U2AF65 and flag-Atx1 82Q plasmids (bar 6). Total RNA was isolated from the cells 24 h post transfection and quantitative RT-PCR analysis using the primer sets shown in B was carried out. The histogram shows the ratios between the isoform levels (py/PY). Values are mean ± standard deviation from four experiments. **§§** indicates P<0.01 GFP-U2AF65 and flag-30Q-Atx1 *vs* GFP-U2AF65 alone; * indicates P<0.05 flag-30Q-Atx1 *vs* empty pCMV vector. Statistical significance was evaluated with One-way analysis of variance followed by Turkey's Multiple Comparison Test. D. Expression of proteins in 293T cells transfected with reporter minigene, U2AF65 and Atx1 constructs. Lanes 1 to 6 correspond to bars 1 to 6 shown in C. Cell extracts were prepared 24 h after transfection and the proteins were analysed by Western blotting with indicated antibodies. Molecular weight markers are indicated on the left.

To test whether association of Atx1 with U2AF65 is splicing-related, we carried out a splicing assay using the reporter pyPY mini-gene, which was previously shown to give rise to a primary transcript incorporating two alternative 3′ sites associated with a weak or strong polypyrimidine tract [35]. In agreement with the previous study, transfection of 293T cells with the pyPY mini gene construct and subsequent RT-PCR analysis revealed three bands: a top band corresponding to the primary unspliced transcript, a middle band resulting from the use of the proximal 3′ weak (py) splice site, and a bottom band from the strong distal 3′ splice site (PY) (**Figure 4A** **, lane 1**). As previously observed [35], over-expression of U2AF65 enhances the splicing from the proximal weak splice site (**Figure 4A** **, lane 2**).

We investigated whether Atx1 might have an effect on U2AF65 mediated splicing. To this end we transfected 293T cells with the pyPY minigene and Atx1 in the presence and in the absence of U2AF65. Total RNA was isolated from the cells 24 h post transfection and quantitative RT-PCR analysis was carried out using specific primers to amplify the two spliced isoforms (**Figure 4B**). In each case, we first analyzed the ratio between the py and PY isoforms, taking the cells singly transfected with the pyPY minigene and empty vectors as reference (**Figure 4C** **, bar 1**). In agreement with the previous report [35], over-expression of U2AF65 was found to enhance the usage of the proximal 3′ weak splice site, resulting in a significant increase in the pyPY ratio (**Figure 4C** **, bar 2**). In cells transfected with the reporter minigene and either non-expanded or expanded Atx1 (**Figure 4C** **, bars 3 and 4**), the py/PY ratio obtained was more than the value obtained when the reporter minigene was co-transfected with empty vectors (**Figure 4C** **bar 1**). Co-transfection of U2AF65 with Atx1 was found to further enhance the splicing from the weak proximal site (**Figure 4C** **, bars 5 and 6**). Further pairwise comparison of subgroups (i.e, control versus cells over-expressing Atx1 constructs, and U2AF65 over-expressing cells versus cells over-expressing both U2AF65 and Atx1) revealed that only non-expanded Atx1 has a significant effect on pyPY splicing (**Figure 4C** **bar 3 and 5**).

Expression of U2AF65 and Atx1 constructs in transfected 293T cells with reporter minigene was compared by Western blot analysis (**Figure 4D**). The results indicated similar expression levels.

Taken together these results point towards a positive effect of Atx1 over-expression on U2AF65-mediated splicing.

### 14-3-3 Prevents U2AF65 UHM from Binding to S776 Phosphorylated Atx1

Since S776 phosphorylated Atx1 binds also the 14-3-3 protein [17], we compared the affinity of this interaction. We used a peptide C-terminally extended as compared to the one previously designed to probe interaction with U2AF65 UHM so to include P778 and encompass the complete 14-3-3 ‘mode II’ recognition motif [31] (Atx1_pS_ULM_PE, residues 769–779). We compared the affinities of this peptide with the non-phosphorylated version (Atx1_S_ULM_PE) and with the shorter Atx1_pS_ULM peptide. Among the nine highly homologous 14-3-3 isoforms, expanded Atx1 has been found to interact *in vivo* with ε, ζ, η, γ, and β isoforms [17]. We chose to work with isoform ζ (14-3-3ζ) since it is present in high concentrations in the neuronal nucleus of all the major affected areas in SCA1 brains [36].

**Figure 5:**
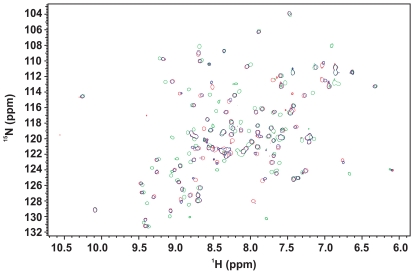
Testing competition of Atx1_pS_ULM_PE binding to 14-3-3ζ and U2AF65_UHM. ^15^N labeled U2AF65_UHM saturated with unlabelled Atx1_pS_ULM_PE was titrated with unlabelled 14-3-3ζ. The HSQC spectra of U2AF65 UHM in its free form (red), saturated with a 2 fold molar excess of Atx1_pS_ULM_PE (green) and saturated with a 1∶1 14-3-3ζ/Atx1_pS_ULM_PE mixture in a 2 fold molar excess to U2AF65 UHM (blue) are compared.

The K_d_ between 14-3-3ζ and Atx1_pS_ULM_PE is two orders of magnitude smaller than the value measured for U2AF65 UHM (**Table 1**) indicating that 14-3-3ζ binding of S776 phosphorylated Atx1 could prevent it from binding to UHMs domains. We probed competition of the two proteins for the Atx1_pS_ULM_PE peptide by NMR titrating unlabelled 14-3-3ζ to ^15^N labeled U2AF65 UHM saturated with unlabelled Atx1_pS_ULM_PE. The HSQC spectrum of free U2AF65 UHM is restored by addition of a 1∶1 ratio of 14-3-3ζ/Atx1_pS_ULM_PE, indicating that U2AF65 has been displaced by 14-3-3ζ (**Figure 5**).

Phosphorylation of S776 was confirmed to be an absolute requirement for binding to 14-3-3, while absence of P778 in Atx1_pS_ULM, a residue highly conserved in Atx1 and in both 14-3-3 ‘mode I’ and ‘mode II’ motifs, only slightly decreased the affinity, still leaving the K_d_ for 14-3-3ζ one order of magnitude lower than for U2AF65. We also tested by ITC the binding of a S776A-mutated peptide (Atx1_A_ULM_PE) to U2AF65 UHM, since it has been shown that Q82-expanded Atx1 carrying a S776A mutation still localizes in the nucleus in transfected cells and in Purkinje cells of transgenic mice but fails to form nuclear inclusions [16]. We confirmed that S776A does not affect substantially the ability of Atx1 to recognize UHM domains and that S776A binds U2AF65 UHM with affinities comparable to the wild-type.

### Atx1 Also Recognizes the Regulatory Splicing Factor SPF45 through a ULM-UHM Interaction

We then reasoned that UHM/ULM interactions might be important also to recognize SPF45, another Atx1 interacting protein which also contains a UHM. To check this hypothesis we extended our ITC studies to this protein (**Table 1**). We observed that Atx1_S_ULM_PE binds SPF45 UHM with lower affinity than U2AF65 UHM but, similarly to what observed with U2AF65, S776 phosphorylation weakens the interaction although to a lower extent. Our in vitro findings using isolated Atx1_S_ULM_PE and SPF45 UHM indicate weak negative rather that positive regulation of the interaction by S776 phosphorylation, in contrast with what observed by co-immunoprecipitation assays from lysate of co-transfected HEK293T cells [18]. These experiments were however carried out using an S776D mutant which should mimic phosphorylated Atx1. To investigate the reasons of these apparently contradictory data, we measured the affinity of both U2AF65 and SPF45 UHMs and 14-3-3ζ for a S776D peptide (Atx1_D_ULM_PE). No binding to 14-3-3ζ was detected, not even when titration was performed at a 0.22 mM 14-3-3ζ concentration, indicating that aspartate does not properly mimic S776-phosphorylation. Conversely, a two-order of magnitude increase of affinity to SPF45 UHM was observed, while affinity to U2AF65 UHM was not significantly affected. Taken together, these findings confirm that S776 phosphorylation plays a limited and direct role in down-regulating Atx1 binding to UHMs and fully explain the differences observed *in vivo* with the S776D mutant [18].

### Modeling of the Complex Allows Rationalization of the Results

To rationalize our results we analyzed the structures of the other UHM/ULM complexes of U2AF65/SF1 (PDB code 1opi, [23]) and SPF45/SF3b155 (2peh, [21]). In the U2AF65 UHM/SF1 ULM of 1opi, position W+1 of the SF1 ULM, which corresponds to S776, is occupied by an asparagine that is fully exposed to the solvent (46 Å^2^) and does not form contacts with U2AF65. In the SF3b155 ULM5 of 2peh, the same position is occupied by an aspartate. This negatively charged residue forms a hydrogen bond with the side chain of a spatially close positively charged UHM residue (i.e. R375 of SPF45) and drastically changes the ULM conformation.

**Figure 6:**
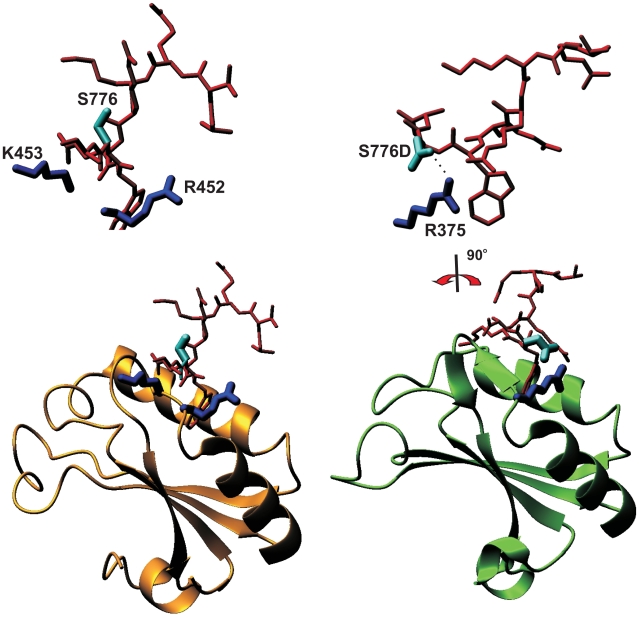
Structural models of U2AF65 and SPF45 in complex with the Atx1 native and S776D mutant ULMs. The structures of 1opi and 2peh were used as homology modeling templates for the complexes of U2AF65 (left) and SPF45 (right), respectively. In the 1opi complex (left), the SF1 ULM peptide (PSKKRRKRSRWNQD) contains an Asn in correspondence to Atx1_ULM S776. The top panel shows a blow-up of the peptide and of R452 and K453 in the same orientation as shown below. The residue does not pack against the protein and is exposed. In the 2peh complex (right), the aspartate in position W+1 of the SF3b155 ULM5 peptide (KRKSRWDETP) hydrogen bond with the spatially close R375 of SPF45. These coordinates are therefore more appropriate to model the structure of the S776D mutant. The top panel shows a blow-up of the hydrogen bond between S776D with the side chain of R375, as rotated by −90 degrees around the y axis.

Using this information, we build homology models of the U2AF65 UHM/Atx1 ULM and SPF45 UHM/Atx1 ULM complexes using 1opi and 2peh as templates (**Figure 6**). When position 776 of Atx1 ULM is occupied by a neutral serine or alanine Atx1_S_ULM_PE and Atx1_A_ULM_PE are likely to adopt a conformation similar to that observed in 1opi, with the residue being exposed therefore explaining why the K_d_s of these complexes are comparable (**Table 1**). When S776 is replaced by an aspartate, this residue will likely behave as in SF1 ULM and form a hydrogen bond with R375 of SPF45 as in 2peh. This explains why we observe a tighter interaction for the SPF45/Atx1_D_ULM_PE.complex. A similar stabilization is not possible when Atx1_ with U2AF65 UHM likely because the mutated D776 is surrounded not only by R452 of U2AF65 (which corresponds to R375 of SPF45) but also by the positive side chains of K453. The three positively charged residues could compete, thus not allowing preferential orientation of the aspartate side-chain for optimal coordination with R452. An aspartate in position W+1 seems to be conserved in all the ULM ligands known to interact with high affinity to UHMs in which the X of the Arg-X-Phe motif is an aromatic (i.e. Y or W) instead of a positively charged residue. These include SPF45 UHM/SF3b155 ULM5 [21], U2AF35 UHM/U2AF65 ULM [21], [22] and PUF60 UHM/SF3b155 ULM1 and ULM3 [37]. Finally, it is clear from both models that a phosphate group at S776 is too bulky to form a hydrogen bond with the protein, thus explaining the slight destabilising effect of phosphorylation.

## Discussion

It is common when studying proteins associated with diseases to consider them mainly under the aspect of how they are linked to pathology. This is particularly the case for polyQ proteins: much has been said about their relationship to aggregation and cellular toxicity but still little is known about their cellular functions when they are in their non-pathologic state. In this study, we have followed a different approach. We focused on the functional role of non-pathologic Atx1 and set out to identify sequence motifs which could provide direct information about the mechanisms involved in recognition.

AAlthough a role in transcriptional regulation, often mediated by interaction of the AXH domain with several transcriptional co-regulators [10]–[14], [38], [39] has also been suggested, independent evidence indicates that Atx1 is an RNA binding protein with a role in processing and/or exporting specific mRNAs to the cytoplasm: Atx1 recognizes RNA homo-polymers [9], [40]; it is linked directly through its interactome to other RNA binding proteins, some of which are splicing factors [18], [26]; Atx1 was shown to recruit the mRNA export factor TAP/NXF1 [41].

We have shown in this study that Atx1 interacts with U2AF65, thus adding a new member to the Atx1 interactome. Atx1 does not co-localize with nuclear speckles, the subnuclear structures in which splicing factors accumulate when not actively forming spliceosomes (reviewed in [42]). A splicing-independent association in extraspliceosomal complexes has recently been described for the splicing factors SF1 and U2AF [43].

Our splicing assays suggest an enhancing effect of Atx1 over-expression on U2AF65-mediated splicing. While the exact implications for the function of Atx1 in the context of splicing remain to be investigated in detail, it is worth noting that only non-expanded Atx1 seems to have a significant effect on U2AF65-mediated splicing suggesting that polyQ expansion could interfere with molecular recognition. Addionally, recruitment or trapping of U2AF65 in the single large nuclear aggregates of SCA1 affected neurons could have a detrimental effect on splicing.

Even more relevant for understanding the functions of native Atx1 is the identification of the motif that mediates the interaction: the presence of a ULM in the C-terminus of Atx1 strongly supports an involvement of the protein in pre-mRNA splicing and a direct involvement in the complex and highly dynamic macromolecular machinery represented by the spliceosome [25], [44]–[46]. This is in line with the reported presence of non-expanded Atx1 in a large protein complex associated in an RNA dependent fashion with the regulatory splicing factor SPF45 (this complex is most likely the early stage complex known as pre-spliceosome although no attempt to identify it was made) [18]. Since many of the splicing factors contain ULM, UHM or both motifs, our results point directly to a participation of Atx1 to the network of multiple and competing interactions linking the splicing factors.

Further complexity to the overall picture is added by the presence of overlapping functional sites in Atx1. Two motifs overlap with ULM, the NLS and the 14-3-3 phospho-S776 ligand motif, known to be essential requirements for the functions of the protein and for development of disease. This prompted us to check the effect of phosphorylation on interaction and to compare our results with a previous study which describes binding of Atx1 with SPF45 by two-hybrid screen and co-immunoprecipitation from mammalian cells [18]. Because of the techniques used, the authors made the *a priori* reasonable assumption that the effect of phosphorylation could be mimicked or abolished by mutation of S776 to an aspartate and to an alanine respectively. They demonstrated that both wild-type and S776D Atx1 interact with SPF45 and that the interaction is strengthened for S776D but abolished when using a S776A mutant. These data are in some agreement with our *in vitro* binding assays but as compared to the non-phosphorylated Atx1 ULM, the phosphorylated peptide binds with lower affinity. This indicates that aspartate does not have the correct properties to mimic a phosphate group, likely because it is smaller, unbranched and with a lower charge density, as further confirmed by observing that the S776D mutant peptide is not recognised by 14-3-3ζ.

S776 is therefore the molecular switch between alternative interactions: when non-phosphorylated, non-expanded Atx1 will be involved through its ULM in a vast network of interactions with splicing factors and contribute to the spliceosome. Phosphorylated Atx1 will bind instead 14-3-3. Phosphorylated Atx1 also recognises other cellular partners as shown by the evidence that the interactions with the transcriptional repressor Capicua (CIC) which targets the AXH domain of Atx1 [12] and other proteins involved in transcriptional regulation, such as the MEF2-HDAC4 complex [38], are weakened in the S776A mutant.

How do these findings tell us something about disease and how do interactions and signal overlap affect SCA1? It has been suggested that this pathology could be the result of the balance between a gain-of-function due to enhancement of interaction with SPF45 and a partial loss-of-function due to weakening of interaction with CIC. A different, non-mutually exclusive possibility is that phosphorylation causes Atx1 to interact with a substantially different network of nuclear proteins than non-phosphorylated Atx1 [6]. Our findings that Atx1 binding to UHMs is modulated in a posphorylation-dependent manner due to the intervention of 14-3-3 open a scenario that would merge the two hypotheses.

**Figure 7:**
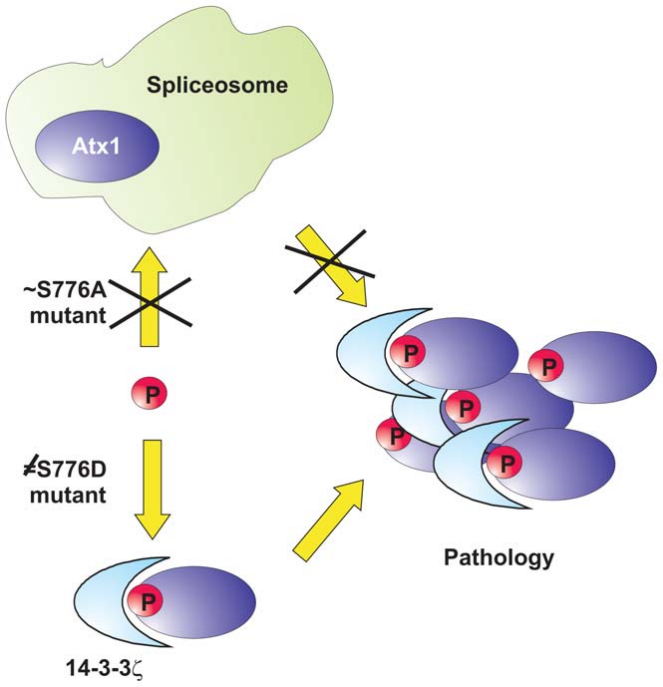
Schematic model of the cellular interactions formed by Atx1 and the role that phosphorylation plays in pathology. S776 phosphorylation would be at the crossing point of two different pathways. When Atx1 (blue oval) is not phosphorylated, it interacts with the spliceosome (light blue shape) and is protected from aggregation. Phosphorylated Atx1 has higher affinity to 14-3-3 proteins but is not protected against self-association. In vitro, the Ala mutant mimics correctly the properties of non-phosphorylated Atx1, whereas the Asp mutant, which has properties very different from the phosphorylated sequence, is not recognized by 14-3-3.

We have demonstrated that the S776A mutant peptide is able to recognize U2AF65 with affinity comparable to that of the native ligand sequence. This indicates that non-phosphorylated expanded Atx1, which does not manifest a SCA1 phenotype as we know from the S776A mutant [16], still recognizes UHM-containing nuclear factors despite being unable to bind 14-3-3. We suggest (**Figure 7**) that participation of Atx1 in these interactions plays a protective role against self-association of the protein induced by polyQ expansion: the spliceosome is a large and dynamic machinery composed of five major small nuclear ribonucleoprotein particles including U2 of which U2AF is an auxiliary factor, and more than three hundred non-snRNP protein splicing factors [25], [44]–[46]. Engagement in such an extended protein complex could easily ‘dilute out’ expanded Atx1 and prevent or reduce its self-association to an extent that allows efficient clearance by the machinery of the proteasome pathway. Conversely, when Atx1 is S776-phosphorylated and recruited by 14-3-3, the interaction with the spliceosome complex is hampered. As a consequence, expanded Atx1 becomes available both to self association through regions such as the polyQ stretches and/or the AXH domain [10], and to recognition of other partners. In support of this hypothesis is also the presence of the 14-3-3ζ isoform in protein aggregate deposits observed in different neurological diseases, such as Parkinson and Alzheimer diseases, Huntington and SCA1 itself [47], [48]. The effect is particularly evident in SCA1 where the 14-3-3ζ isoform changes localization from the cytoplasm to the nucleus of affected Purkinje cells [36].

An important point that becomes clear from our studies is the importance of investigating the non pathologic molecular interactions as a strategy to identify mechanisms that prevent aggregation. Also, it is clear that it is far too simplistic to approach SCA1 or other polyQ diseases in terms of a gain or loss-of-function. These concepts are particularly inadequate when function is described in terms of enhanced or reduced interactions and strongly depend on which of the several interactions we may refer to. As remarked in a recent review [49] and as indicated by our results, polyQ proteins take part in a complex and vast network of interactions, to which polyQ aggregation adds another competing pathway.

For complex regulatory systems, the difficult balance of multiple equilibria can only be appropriately described by gaining an overall picture which places each interaction into the bigger frame of all what is known about the individual components [50]. It will be interesting to investigate further the role of different regions of Atx1 in modulating interactions since SPF45 and CIC, although binding to different sites, i.e. ULM and AXH, compete with each other [12], [18].

## Methods

### Protein Preparation

Unlabelled recombinant U2AF65 UHM (residues 369–475) and SPF45 UHM (residues 301–401) were over-expressed in the *E. coli* host strain BL21 (DE3)pLysS in Luria Broth medium using a kanamycin-resistant pETM30 vector with tobacco etch virus (TEV)-cleavable N-terminal His_6_-GST tags. Isotopically ^15^N- and ^13^C/^15^N labelled samples were expressed in minimal (M9) medium containing ^15^N-ammonium sulphate and^ 13^C-glucose as the sole sources of nitrogen and carbon, respectively. The cells were grown at 37°C until an optical density (OD) at 600 nm of 0.6 was reached, then induced with IPTG (0.25 mM) and harvested after three hours. A standard purification protocol was performed using a Ni-NTA agarose column (Qiagen) except that no imidazol was added before elution. The proteins were further purified by FPLC size exclusion chromatography using a prepacked HiLoad 16/60 Superdex^TM^ 75 prep grade column (Pharmacia). Synthetic Atx1 peptides were purchased from Pepceuticals Limited (Nottingham-UK) and used after having been freed from residual trifluoroacetic acid by repeated steps of dissolution in sterile double-distilled water and lyophilization. Protein and peptide concentrations were checked by measuring UV absorbance at 280 nm.

### NMR Experiments

NMR experiments were performed at 295 K on Bruker Avance 600 and Varian Inova 800 spectrometers, both equipped with cryoprobes. Samples of ^15^N labelled U2AF65 UHM and SPF45 UHM used for the NMR experiments were concentrated to 0.2 mM and dialysed either against 20 mM sodium phosphate buffer (pH 6.4), 30 mM NaCl, 5 mM DTT, or 50 mM sodium phosphate buffer (pH 7.0), 150 mM NaCl, 5 mM DTT (for the U2AF65 UHM/14-3-3ζ competition experiment). Titrations were performed with stepwise additions of concentrated stock solutions of Atx1 ULM peptides up to a 2–3 fold molar excess.

### Comparative Modelling

The U2AF65 and SPF45 UHM/Atx1 ULM complexes were modeled by homology using the coordinates of the U2AF65 UHM/SF1 ULM (PDB code 1opi, [23]) and SPF45/SF3b155 ULM5 (2peh, [21]). The Biopolymer module of Insight2 (Accelrys, San Diego) was used to replace the peptide side chains. The obtained models were submitted to 100 cycles of energy minimization to regularize the geometry.

### ITC Measurements

Binding affinities of U2AF65 UHM, SPF45 UHM and 14-3-3ζ with Atx1 ULM ligands were measured at 295 K by a MicroCal Omega VP-ITC (MicroCal Inc., Northampton, USA). All the proteins were dialyzed against 50 mM sodium phosphate buffer (pH 7.0), 150 mM NaCl and 5 mM DTT. Solutions of 0.025–0.4 mM UHM proteins in the cell were titrated by injections of a total of 295 µl of 0.25–3.9 mM peptide in the syringe in 21 aliquots. The concentrations of the 14-3-3ζ and ATX1 ULM ligands were adjusted to 0.012–0.037 mM and 0.1–0.4 mM respectively. Data were processed with the software MicroCal Origin version 5.0 provided by the manufacturer. All dissociation constants were averages of three measurements.

### Confocal Microscopy

For co-localisation experiments, expanded and non-expanded Atx1 were cloned into pBudCE4.1 vectors expressing monomeric Red Fluorescence Protein (RFP). HeLA cells were transfected with expanded or non-expanded Atx1-RFP constructs. Immunofluorescence was carried out as described previously [51]. Briefly, the cells were fixed 24–48 h after transfection using 4.0% paraformaldehyde, permeabilised with 0.2% triton X-100/PBS and probed with anti-U2AF65 monoclonal antibodies (Sigma) followed by FITC conjugated secondary antibodies. For Atx1 localization studies with a nuclear speckle marker, cells were transfected with Atx1-RFP constructs and stained with anti SC-35 monoclonal antibodies (Abcam Plc) followed by FITC conjugated secondary antibodies. After washing with PBS, slides were mounted using Citifluor (Agar Scientific) and analysed by confocal microscopy. Cells were visualised under a Leica laser scanning confocal microscope (TCS-SP1) equipped with a DM-RXE microscope and an argon-krypton laser. Images were acquired as single 0.2 µm trans-cellular optical sections and averaged over 20 scans per frame. Images were acquired sequentially and appropriate emission filter settings and controls were included to minimize bleed-through effects.

### Co-Immunoprecipitation

HeLa cells were plated at equal density and transfected with either flag-tagged Atx1 constructs or with empty pCMV flag vectors. Cells were trypsinised, pelleted and washed with PBS. 5% of the cell pellet was solubilized with SDS sample buffer and subjected to western blot analysis by respective antibodies to check expression levels. The rest of the cells were lysed in TS buffer (20 mM Tris-HCl pH 7.5, 75 mM NaCl) containing 1.0% NP40 and supplemented with protease and RNAse inhibitors. The cleared lysate was diluted 6 times with TS buffer without detergent and incubated with either Atx1 (11NQ, obtained from NeuroMab Facility, University of California, Davis) or U2AF65 (Sigma) monoclonal antibodies and protein A/G agarose at 4°C overnight. Pellets were washed three times with TS buffer. After SDS-PAGE, Atx1 and U2AF65 were detected by immunoblotting with anti-flag and anti-U2AF65 antibodies and peroxidase conjugated Light Chain specific anti-mouse IgG secondary antibodies (Jackson ImmunoResearch). Cerebellum extracts were prepared by homogenizing tissue in TS buffer containing 1.0% NP40 and protease/RNAse inhibitors) using a Polytron PT 3000 homogenizer. Cleared lysates were diluted 6 times with TS buffer without detergent. 500 µl of lysates (1.0 mg protein) were used to perform immunoprecipitation using the anti-Atx1 monoclonal antibody 11NQ. Similar amounts of lysates were used for immunoprecipitation using anti-U2AF65 monoclonal antibodies or a control monoclonal antibody (anti-flag antibody, Sigma). Pellets were washed with TS buffer, resuspended and boiled in sample buffer. Immunoprecipitated samples were analysed by PAGE and Western blot using anti-Atx1 or anti-U2AF65 antibodies.

### Splicing Assay

HeLa cells were transfected with pyPY minigene encoding plasmids. 24 h post transfection, total RNA was prepared from the cells using RNAspin mini RNA purification system (GE HealthCare). For RT PCR analysis, first strand synthesis was carried out using Superscript III qRT-PCR mix (Invitrogen) and the PCR products were separated on agarose gels and detected by ethidium bromide staining. The following primers were used for the PCR: 5′ TGAGGGGAGGTGAATGAGGAG 3′and 5′ TCCACTGGAAAGACCGCGAAG 3′.

To analyse the effect of Atx1 on splicing, 293T cells were plated on 24 well plates and co-transfected with pyPY reporter minigene and GFP-U2AF65 plasmids (both kindly supplied by Dr Juan Valcárcel), in the presence and absence of Atx1 expression vectors. Control cells were transfected with pyPY reporter and appropriate empty vectors. Total RNA was isolated after 24 h as described above. First strand synthesis was carried out using the Quantitect reverse transcription kit (Qiagen). Real-time quantitative PCRs were performed on a Rotor Gene 6000 real-time rotary analyzer (Corbett Research) using SensiMix Plus SYBR kit (Quantace). Annealing and extensions were carried out at 60°C and 72°C, respectively. Absolute copy numbers of the transcripts derived from standard curves were used to generate py/PY ratios. Sequence of the forward primer and the reverse primers used were as follows: forward primer, 5′ AGGCTTTGAGAACCTGTGGA 3′. Distal reverse primer, 5′ CCTCAACCGCGAGCTTGA 3′. Proximal reverse primer, 5′ GAGAGTCATTTCACCTTGA 3′. The PCR products were confirmed by sequencing. Statistical analyses were performed with Prism software (GraphPad software). All values are expressed as mean ± standard deviations from four independent experiments. Statistical analysis was assessed by One-way ANOVA followed by Turkeys Multiple Comparison Test. Probabilities (P) <0.05 were considered significant.

## Supporting Information

Figure S1 ITC profiles for the binding of the Atx1 peptides. In each panel, the raw data relative to sequential injections are shown in the upper plot; the lower plot represents the resulting integrated enthalpy data fit to a single-site binding model (solid line). The curves represent the titration of Atx1_S_ULM_PE, Atx1_pS_ULM_PE and Atx1_D_ULM_PE (from left to right) with U2AF65 (upper panel) and 14-3-3ζ (lower panel).
(1.92 MB TIF)

Figure S2 Some speckle-like formations of U2AF65 are devoid of SC-35. HeLa cells were transfected with GFP-U2AF65. Fixed, permeabilized cells were probed with antibodies against nuclear speckle marker SC-35 followed by TRITC conjugated secondary antibodies. Confocal Microscopy: HeLa cells growing in chamber slides were transfected with GFP-U2AF65 constructs. Cells were fixed and permeabilized and incubated with anti SC-35 monoclonal antibody (Abcam Plc) followed by secondary antibodies conjugated to TRITC. After washing with PBS, slides were mounted using Citifluor (Agar Scientific) and analysed by confocal microscopy. Cells were visualised under a Leica laser scanning confocal microscope (TCS-SP1) equipped with a DM-RXE microscope and an argon-krypton laser. Images were acquired as single 0.2 µm transcellular optical sections and averaged over 20 scans/frame. Images were acquired sequentially and appropriate emission filter settings and controls were included to minimize bleed-through effects. Images were merged using the ImageJ program (NIH, Bethesda). Merged images show the details observed in a single 0.2 µm optical section.
(0.54 MB TIF)

Figure S3 Assessment of CD44 pre-mRNA occupancy. Cells were transfected with GFP-U2AF65, empty pCMV-flag vector, CD44 minigene construct and CAT expression vector (lane1); GFP-U2AF65, flag-expanded Atx1, CD44 minigene construct and CAT expression vector (lane 2) or GFP-U2AF65, flag-unexpanded Atx1, CD44 minigene construct and CAT expression vectors (lane 3). RNP immunoprecipitations were carried out and RT PCR was performed using primers specific for CD44 (top panel). 5% of the cells used for analysis were subjected to western blotting using anti CAT antibodies (bottom panel). RNP Immunoprecipitation: Experiments were carried out as previously described [1]. In short, cells were transfected with GFP-U2AF65 and CD44 minigene constructs (kindly supplied by Dr. Harald König, Karlsruhe) and a CAT expression vector used as an internal transfection control. Atx1 expression vectors were added in the transfection mix to test the effect on pre mRNA occupancy. In vivo crosslinking using 0.1% formaldehyde was carried out as described [2]. 5% of the cross-linked cells were pelleted, solubilized in SDS sample buffer and subjected to western blotting with an anti CAT antibody (Sigma). The rest of the cross-linked complexes were solubilized by sonification. Immunoprecipitation was performed by incubating the lysates with polyclonal rabbit anti-GFP antibody (Abcam) at RT for 90 min. Prior to immunoprecipitation, protein A/G was incubated with 20 units of RNasin (Promega) for 10 min. Immunoprecipitates were washed four times with RIPA buffer. Precipitated complexes were de-cross-linked in 100 µl 50 mM Tris-HCl pH 7.0, 1% SDS, 5 mM EDTA, 10 mM DTT at 70°C for 45 min and RNA was extracted using Pure Link RNA purification system (Invitrogen) followed by DNase digestion. Half of the RNA was reverse transcribed by random priming. The following primers were used as forward and reverse primers, TCATTATGACAGTTGCCACCAG and TACTTGTGCTTGTAGCATGTGG, for detecting the pre-mRNA fragment from the pETv5 minigene (607 bp in length). Thirty-five PCR cycles (95°C for 1 min; 55°C for 1 min; and 72°C for 1.5 min) were performed for amplification of fragments from the transfected minigene. References 1. Tisserant A, Konig H (2008) Signal-regulated Pre-mRNA occupancy by the general splicing factor U2AF. PLoS ONE 3: e1418. 2. Niranjanakumari S, Lasda E, Brazas R, Garcia-Blanco MA (2002) Reversible crosslinking combined with immunoprecipitation to study RNA-protein interactions in vivo. Methods (San Diego, Calif) 26: 182–190.
(0.18 MB TIF)
