# Supplementary figures and images for: Phosphorylation of S776 and 14-3-3 Binding Modulate Ataxin-1 Interaction with Splicing Factors
Source: PLoS One. 2009 Dec 23;4(12):e8372. doi: 10.1371/journal.pone.0008372 (PMC2791216; doi:10.1371/journal.pone.0008372)

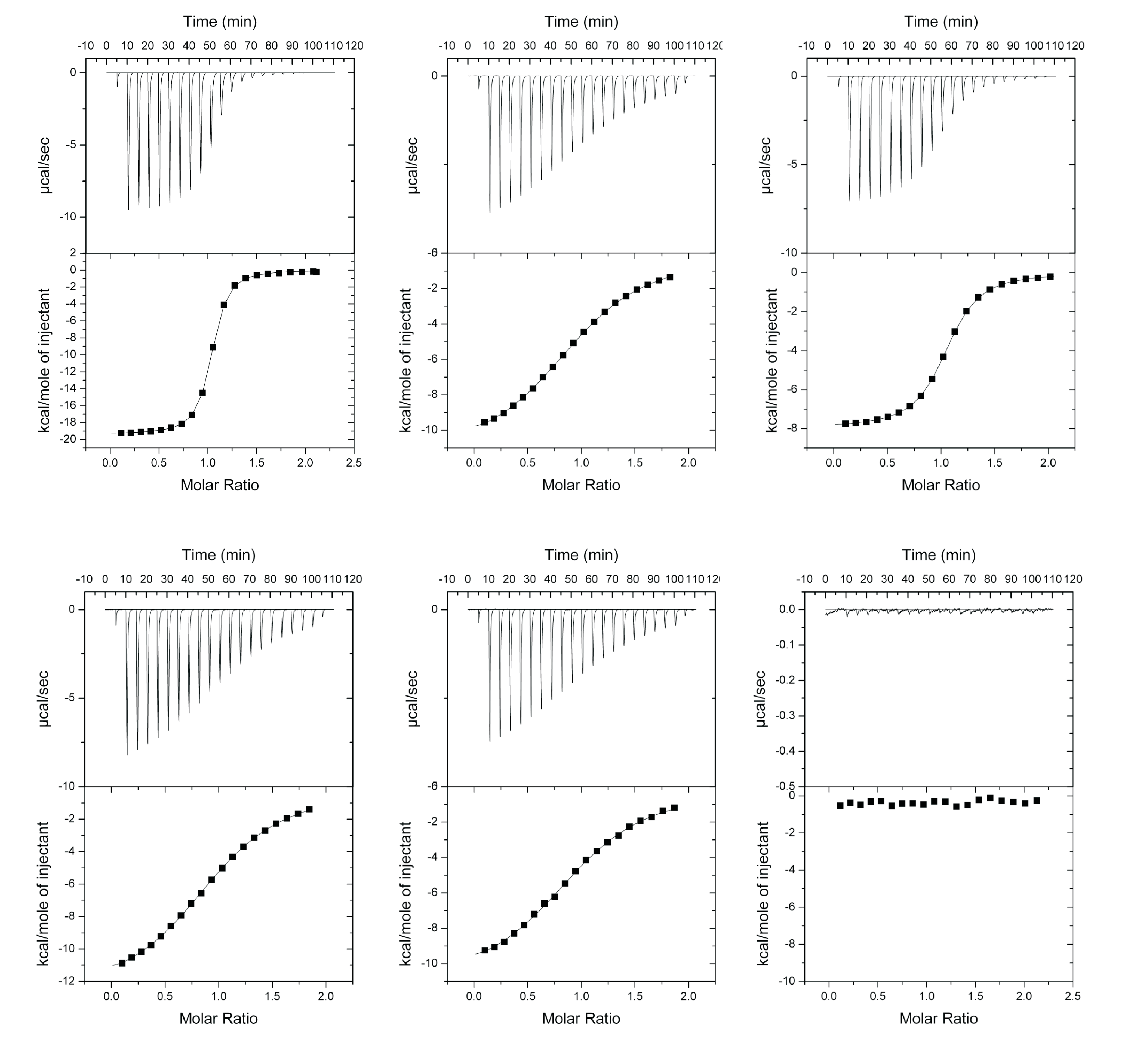

Supplement: Figure S1 — ITC profiles for the binding of the Atx1 peptides. In each panel, the raw data relative to sequential injections are shown in the upper plot; the lower plot represents the resulting integrated enthalpy data fit to a single-site binding model (solid line). The curves represent the titration of Atx1_S_ULM_PE, Atx1_pS_ULM_PE and Atx1_D_ULM_PE (from left to right) with U2AF65 (upper panel) and 14-3-3ζ (lower panel). (1.92 MB TIF) [file pone.0008372.s001.tif]

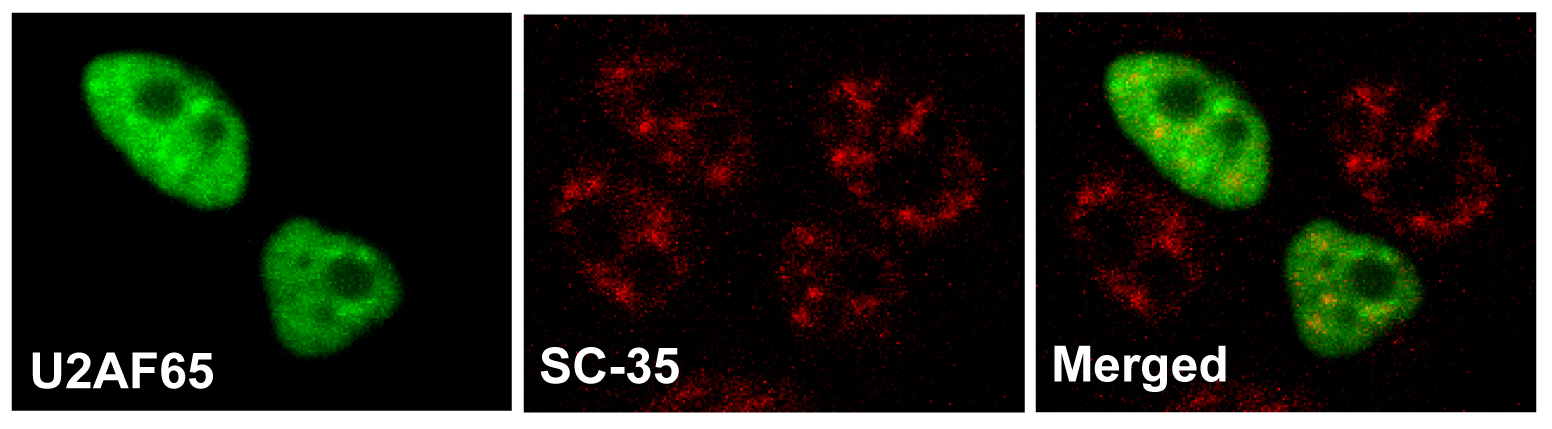

Supplement: Figure S2 — Some speckle-like formations of U2AF65 are devoid of SC-35. HeLa cells were transfected with GFP-U2AF65. Fixed, permeabilized cells were probed with antibodies against nuclear speckle marker SC-35 followed by TRITC conjugated secondary antibodies. Confocal Microscopy: HeLa cells growing in chamber slides were transfected with GFP-U2AF65 constructs. Cells were fixed and permeabilized and incubated with anti SC-35 monoclonal antibody (Abcam Plc) followed by secondary antibodies conjugated to TRITC. After washing with PBS, slides were mounted using Citifluor (Agar Scientific) and analysed by confocal microscopy. Cells were visualised under a Leica laser scanning confocal microscope (TCS-SP1) equipped with a DM-RXE microscope and an argon-krypton laser. Images were acquired as single 0.2 µm transcellular optical sections and averaged over 20 scans/frame. Images were acquired sequentially and appropriate emission filter settings and controls were included to minimize bleed-through effects. Images were merged using the ImageJ program (NIH, Bethesda). Merged images show the details observed in a single 0.2 µm optical section. (0.54 MB TIF) [file pone.0008372.s002.tif]

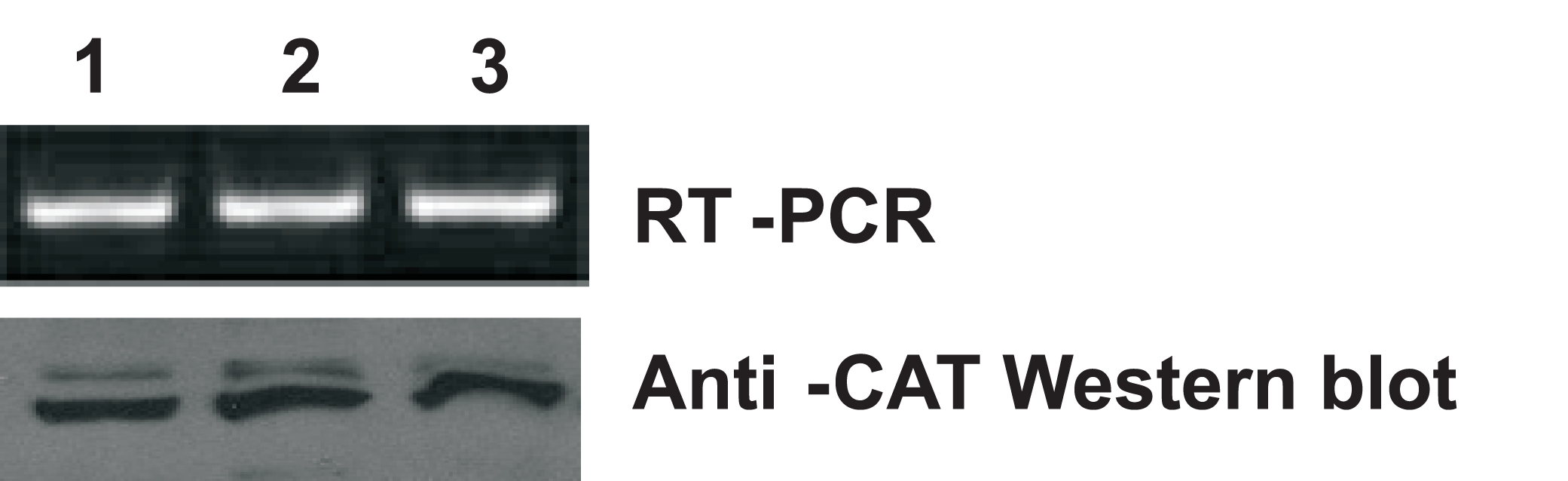

Supplement: Figure S3 — Assessment of CD44 pre-mRNA occupancy. Cells were transfected with GFP-U2AF65, empty pCMV-flag vector, CD44 minigene construct and CAT expression vector (lane1); GFP-U2AF65, flag-expanded Atx1, CD44 minigene construct and CAT expression vector (lane 2) or GFP-U2AF65, flag-unexpanded Atx1, CD44 minigene construct and CAT expression vectors (lane 3). RNP immunoprecipitations were carried out and RT PCR was performed using primers specific for CD44 (top panel). 5% of the cells used for analysis were subjected to western blotting using anti CAT antibodies (bottom panel). RNP Immunoprecipitation: Experiments were carried out as previously described [1]. In short, cells were transfected with GFP-U2AF65 and CD44 minigene constructs (kindly supplied by Dr. Harald König, Karlsruhe) and a CAT expression vector used as an internal transfection control. Atx1 expression vectors were added in the transfection mix to test the effect on pre mRNA occupancy. In vivo crosslinking using 0.1% formaldehyde was carried out as described [2]. 5% of the cross-linked cells were pelleted, solubilized in SDS sample buffer and subjected to western blotting with an anti CAT antibody (Sigma). The rest of the cross-linked complexes were solubilized by sonification. Immunoprecipitation was performed by incubating the lysates with polyclonal rabbit anti-GFP antibody (Abcam) at RT for 90 min. Prior to immunoprecipitation, protein A/G was incubated with 20 units of RNasin (Promega) for 10 min. Immunoprecipitates were washed four times with RIPA buffer. Precipitated complexes were de-cross-linked in 100 µl 50 mM Tris-HCl pH 7.0, 1% SDS, 5 mM EDTA, 10 mM DTT at 70°C for 45 min and RNA was extracted using Pure Link RNA purification system (Invitrogen) followed by DNase digestion. Half of the RNA was reverse transcribed by random priming. The following primers were used as forward and reverse primers, TCATTATGACAGTTGCCACCAG and TACTTGTGCTTGTAGCATGTGG, for detecting the pre-mRNA fragment from the pETv5 mini [file pone.0008372.s003.tif]
